# Assessing the Roles and Responsibilities of Informal Caregivers from the Perspective of Adult Patients in Saudi Arabia: A Cross-Sectional Study

**DOI:** 10.3390/healthcare13091038

**Published:** 2025-05-01

**Authors:** Saja H. Almazrou, Shiekha S. Alaujan, Nouf F. AlSaad

**Affiliations:** 1 Clinical Pharmacy Department, College of Pharmacy, King Saud University, Riyadh 11362, Saudi Arabia; salaujan@ksu.edu.sa; 2 Royal Commission Hospital, Jubail 31961, Saudi Arabia; noof.f.alsaad@gmail.com

**Keywords:** informal caregivers, healthcare recipients, roles, challenges, Saudi Arabia

## Abstract

**Objectives:** This study aim to determine the characteristics, roles, responsibilities, and challenges of informal caregivers for adult patients in Saudi Arabia. **Methods**: Adult patients who have informal caregivers were invited to participate in a cross-sectional study. The inclusion criteria were patients who were 18 years old or older and permanent Saudi residents. A self-administered online questionnaire was used to identify patients’ demographics, roles, responsibilities, and care challenges. Data collection lasted four months. Percentages, means, and standard deviations were reported in the analysis. **Results:** The study included 276 participants, mostly female (68.8%), with a mean age of 55.21 years (SD = 20.3). Over half were married (56.2%) and not employed (81.9%). Common chronic diseases were diabetes and hypertension, with 55.8% using up to five medications. Caregivers were mainly sons or daughters (62%) living with the patient (84.1%). The top caregiver tasks were escorting patients to appointments (63.4%), scheduling doctor appointments (60.1%), and tracking medication refills (59.4%). Common challenges included caregivers lacking time (45.3%), inconsistent care (35.9%), financial constraints (27.5%), and caregivers missing doses (27.9%). The top not encountered challenges were inappropriate medication storage (78.3%), communication barriers (74.3%), improper disposal of injections (72.5%), medication management errors (71.4%), and lack of empathy (70.3%). **Conclusion:** This study highlights the vital role of informal caregivers in managing chronic illnesses in Saudi Arabia. Informal caregivers face challenges such as time constraints and financial limitations. The findings emphasize the need for better support systems, including training programs and improved access to healthcare resources, to enhance care quality for patients.

## 1. Introduction

Informal caregivers play a crucial role in providing support to individuals with various needs, particularly older adults, encompassing a wide range of tasks that are essential for their care recipients’ well-being. Understanding these responsibilities is vital for recognizing the impact of informal caregiving on caregivers and the healthcare system. Key responsibilities include physical assistance, in which caregivers often help with daily activities such as mobility, medication management, and personal hygiene, which are critical for maintaining their care recipient’s health [1]. They also serve as navigators in healthcare systems, helping older adults access necessary services and advocating for their needs [2]. Informal caregivers also provide emotional and social support, which are essential for their care recipients’ mental well-being [3]. Additionally, they frequently coordinate among various healthcare providers and services to ensure continuity of care and address any gaps in service delivery [2]. The financial impact of caregiving is substantial, with caregivers in the United States delivering approximately 37 billion hours of care each year, amounting to an estimated value of USD 470 billion. This underscores the vital contribution caregivers make to the healthcare system [4].

In Saudi Arabia, patients often rely on family members or domestic workers (e.g. housemaids) to assist them with a wide range of tasks, including those related to their health [5]. A national study revealed that nearly half of all informal caregivers in the country are housemaids, highlighting the significant role they play in the caregiving landscape. Furthermore, the same study found that 66% of patients depend on their caregivers for critical tasks such as medication management. This reliance on informal caregivers underscores the importance of identifying and understanding this group, as it can help pinpoint individuals who are highly dependent on caregivers and ensure they receive the appropriate support they need.

Although substantial international literature has explored the roles and challenges of informal caregivers, much of this research is situated in Western contexts and focuses primarily on family caregivers [6,7,8]. These studies may not fully reflect the caregiving dynamics in countries like Saudi Arabia, where domestic workers play an unusually prominent role in providing health-related care. In addition, most caregiving literature investigates the views of the caregivers, and limited studies explore the caregiving process from the healthcare recipients’ perspective. Understanding the perspectives of care recipients is essential, as they may differ significantly from the caregiver’s own perceptions. The voices of care recipients can inform more balanced and patient-centered policy and service development [9].

Despite the critical role of informal caregivers in Saudi Arabia, research on this topic remains limited. Existing studies have primarily focused on the burden experienced by family caregivers [10,11] or have described the demographic characteristics of caregivers and care recipients, along with the scope of care they provide [5,12]. For instance, some studies have explored the emotional, physical, and financial challenges faced by family members who take on caregiving roles, while others have outlined the general responsibilities of caregivers in the Saudi context. However, none of these studies have specifically addressed the tasks and challenges associated with involving informal caregivers—such as housemaids or other domestic workers—in the provision of healthcare to patients.

This gap in the literature highlights the need for a more focused investigation into the roles, responsibilities, and challenges of informal caregivers in Saudi Arabia. By doing so, we can gain a deeper understanding of how these caregivers contribute to the healthcare system, the specific tasks they perform, and the difficulties they encounter. Such insights are crucial for developing targeted interventions and support systems that can improve the quality of care provided to patients and alleviate the burdens faced by informal caregivers.

Therefore, this study aims to determine the roles, responsibilities, and challenges of informal caregivers in Saudi Arabia. By shedding light on these aspects, we hope to contribute to a more comprehensive understanding of the caregiving landscape in the country and inform policies and practices that better support both caregivers and the patients who depend on them. This research is particularly timely and relevant, given the growing reliance on informal caregivers in Saudi Arabia and the need to ensure that their contributions are recognized and supported within the healthcare system.

## 2. Methodology

### 2.1. Study Design

A cross-sectional study was conducted in Saudi Arabia to investigate the experiences and perspectives of healthcare recipients toward caregivers. The study utilized an online data collection method through a self-reported questionnaire. The survey was distributed via Twitter, a social media platform, to reach a broad audience efficiently. Data collection spanned four months, ensuring a sufficient sample size and diversity in responses. 

### 2.2. Participants

A convenient sample of healthcare recipients were invited. The use of a convenience sample was justified by the need for rapid data collection and the accessibility of participants through social media platforms like Twitter. The inclusion and exclusion criteria were designed to ensure that the study focused on the experiences of adult healthcare recipients in Saudi Arabia who rely on informal caregivers, thereby providing insights into the dynamics of caregiving within familial or domestic settings. The inclusion criteria for the study required healthcare recipients to be living in Saudi Arabia, aged 18 years or more, and report having a caregiver, either a family member or a domestic worker, at the time of initial screening. Conversely, the exclusion criteria specified that healthcare recipients living outside Saudi Arabia; independent patients; informal caregivers for children; and those with formal caregivers, such as paid nurses, were not eligible to participate in the study.

### 2.3. Data Collection/Data Source

#### Recruitment

Twitter was used as the main source to approach the target healthcare recipients. We created an account for this study, highlighting the research problem and potential implications. Then, we posted the survey on a regular basis. We contacted several national accounts related to chronic disease organizations and governmental bodies in health to post the survey to their accounts.

### 2.4. Instrument

SurveyMonkey [13] was used to create the online survey. The first page gave a brief description of the study’s aim. Then, consent to participate in the study was required. To ensure that we targeted the right sample, we asked a few screening questions, which included the country of current residence, the need for caregivers (family or paid domestic worker), and the participant’s age. If the participant passed the screening section, the survey questions started appearing afterward. If the participants did not pass some of the screening questions, the survey ended at this point.

The instrument used in this study consisted of three sections. The first section captured the healthcare recipients’ demographic data. The second section was a checklist intended to assess the level of caregiver involvement in providing care to the healthcare recipients. The expected caregivers’ tasks were derived from published studies [14]. The third section assessed reasons for needing assistance and health-related challenges arising from the caregivers’ provision of the patients’ healthcare. The total number of roles and responsibilities was 16 and the total number of challenges associated with caregiving was 12. Three Likert scales were used for the second and third sections.

The survey was reviewed by three professionals who determined its validity. It was utilized in a five-participant pilot study to check the clarity and applicability. The study survey is given in Supplementary File S1.

Statistical Analysis: Statistical analyses were conducted using SPSS 22. Descriptive analysis was conducted to describe the data. Numbers and percentages were used to present categorical data and means, and standard deviation was used to represent continuous data. This study was exploratory in nature and did not involve regression or multivariate analyses, as its primary objective was to describe and understand the roles, responsibilities, and challenges of informal caregivers in Saudi Arabia.

### 2.5. Ethical Considerations

Participation in the study was voluntary. Participants’ name were not revealed. The collected data were kept private. The consent page was displayed on the first page of the questionnaire to all participants, clearly indicating the purpose of the study. No incentives or rewards were given to the participants, and their anonymity and confidentiality were guaranteed. Ethical approval was obtained from King Fahad Medical City (IRB number 20-436E).

## 3. Results

The number of study participants was 276 (68.8% female), with a mean age of 55.21 (SD = 20.3), and the majority had a secondary or low educational level (39.6%) or university educational level or higher (31.9%) (Table 1). More than half of the sample were married (56.2%) and not employed (81.9%). Ninety-seven percent were Saudis from the central region (62.7%), living in an urban region (88%) with a spouse or children (43.8%). Regarding chronic diseases, 62.7% and 46% reported having diabetes and hypertension, respectively (Table S1), and used up to five medications (55.8%) for their chronic illnesses. Most of the patients (68.5%) reported using medications that require caregivers’ assistance, such as injections (27.9%) and eye and ear drops (19.2%).

The majority of caregivers were sons or daughters, (62%), lived together with the patient (84.1%), and provided care a maximum of 8 h weekly (55.1%) for more than two years (60.1%).

**Figure 1:**
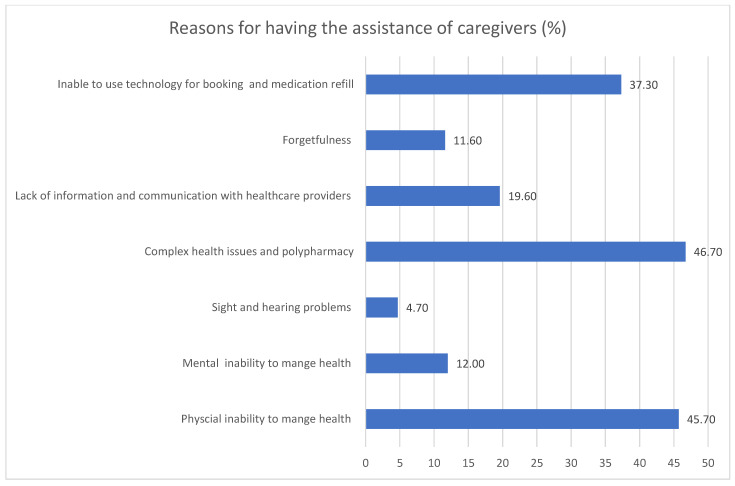
Reasons participants reported for having caregivers’ assistance, as percentages.

Patient-reported reasons for the need for caregiver assistance are illustrated in Figure 1. The most common were having complex health issues and polypharmacy (46.7%), followed by the presence of physical inability to manage health (45.7%), the inability to use technology for booking appointments, and refilling medications (37.3%).

Caregivers’ roles and responsibilities were reported by 248 participants. The top three rated roles and responsibilities were escorting the patient to an appointment (63.4%), followed by scheduling doctors appointments (60.1%), and keep tracking of medication refills (59.4%) whereas injecting medication into patients (40.6%), wound ostomy care (42.8%), and helping the patient bathe and maintain personal hygiene (49.6%) were the bottom three rated roles and responsibilities (Table 2).

The challenges encountered during care (*n* = 248) are presented in Table 3. Patients reported always or sometimes having challenges with the caregiver because they has no time (45.3%), inconsistent care because the caregiver has other responsibilities (35.9%), the patient being unable to afford a paid domestic worker (27.5%), and the caregiver missing doses (27.9%). Of the most common not encountered challenges, the caregiver stored medication (e.g., insulin) inappropriately (78.3%), had difficulty communicating due to a language barrier (74.3%), inappropriately disposed of injections (72.5%), made an error during medication management (e.g., wrong medication or wrong frequency/dose) (71.4%), or lacked empathy (70.3%).

## 4. Discussion

The study highlights the demographics of healthcare recipients who require informal caregivers in Saudi Arabia and the caregivers’ roles, responsibilities, and challenges when they provide care to the recipients. The sample involved a diverse group of participants, primarily middle-aged females with various educational backgrounds, most of whom were married and unemployed. The majority reported chronic health conditions, often managing multiple medications. The caregivers, primarily adult children, lived with the healthcare recipients and contributed several hours of care each week. Healthcare recipients emphasized their need for assistance due to complex health issues and physical limitations. Key caregiver tasks included accompanying patients to appointments and managing scheduling, and common challenges faced included time constraints, inconsistent care, and difficulties in medication management, alongside issues with medication storage and communication barriers.

The use of technology was one of the main reasons for having informal care assistance in the current study. Our results were consistent with a published systematic review that highlighted that age-related physical limitations, such as hearing and poor vision; cognitive disorders, such as poor memory; and limited learning skills were the main barriers to adopting healthcare technologies [15].

Escorting patients to appointments and scheduling were reported in our study as caregiver tasks. This aligned with several studies that reported similar practical caregiver tasks, such as contacting home care services, scheduling appointments, and providing transportation [6,7,8]. They also kept records of test results and handled multiple medications for older adults with complex needs [6,7,8].

According to the current study’s results on the roles and responsibilities of caregivers, tracking medicine refills, preparing pill boxes, and administering medication to patients were the most reported tasks. This aligns with Reinhard et al., who reported that more than one third of informal caregiver respondents helped with various medication management responsibilities [16].

Caregivers have reported making medication errors, particularly in the storage and administration of medications. These errors include losing pills, administering medications at incorrect times, and missing doses [17]. A study showed that one in eight caregivers involved in medication management acknowledged making mistakes in administering medications [18]. In the current study, a minority of healthcare recipients reported having issues with their caregiver making a mistake during medication management (e.g., incorrect medicine or inappropriate frequency/dosage) or missing a medication dose. In addition, a small number of healthcare recipients reported that their caregiver lacks specialized skills needed (e.g., using injection or nebulizers).

### 4.1. Limitations

The study has several limitations that should be considered. First, the use of a convenient sample and recruitment through Twitter may introduce sampling bias because the sample might not represent the entire population of healthcare recipients in Saudi Arabia. Additionally, the reliance on self-reported data can lead to response bias, with participants potentially providing inaccurate or socially desirable answers. In addition, technical issues were encountered when the participants filled the online questionnaire and led to incomplete surveys by some respondents.

### 4.2. Implication

This study’s results have several important implications. First, the high prevalence of chronic diseases, such as diabetes and hypertension, among the healthcare recipients highlights the significant burden of these conditions in the population. The reliance on caregivers for medication administration and other health-related tasks underscores the critical role caregivers play in managing chronic illnesses. The findings also indicate that many caregivers are family members, primarily sons or daughters, who provide care despite having other responsibilities, which can lead to inconsistent care and missed doses. The reported challenges, such as caregivers lacking time and the ability to afford paid domestic workers, suggest a need for better support systems for healthcare recipients and caregivers. Additionally, the low incidence of certain problems, such as inappropriate medication storage and communication barriers, suggests that some aspects of caregiving are being managed effectively. However, the overall findings emphasize the need for targeted interventions to support caregivers, improve medication management, and address the specific needs of healthcare recipients with chronic illnesses. This could include training programs for caregivers, better access to healthcare resources, and policies to alleviate the financial burden on families [2,15].

In the Saudi Arabian context, where caregivers often include domestic workers, the caregiving process may be influenced by varying levels of health literacy, education, and language barriers. These factors can hinder effective communication, understanding of medical instructions, and safe medication practices, making culturally and linguistically tailored training programs essential.

This study provides a foundation for future investigations into the quality and safety of informal caregiving, especially when non-family members are involved. Future research should explore the perspectives of caregivers themselves to identify training needs and to develop targeted interventions that address role overload, time constraints, and knowledge gaps. Longitudinal studies are also warranted to evaluate how caregiver-related challenges impact patient outcomes over time. In addition, future studies should examine the influence of demographic factors—such as age, gender, and education level—on caregivers’ roles and responsibilities

## 5. Conclusions

In conclusion, this study highlights the significant roles, responsibilities, and health challenges of informal caregivers in managing the health of patients from the perspective of their recipients in Saudi Arabia. The findings reveal that caregivers, primarily family members, are heavily involved in various health-related tasks, including medication management and attending medical appointments. However, caregivers face numerous challenges, such as time constraints, inconsistent care due to other responsibilities, and financial limitations. These issues underscore the need for enhanced support systems for healthcare recipients and caregivers. Targeted interventions, such as caregiver training programs, improved access to healthcare resources, and policies to reduce the financial burden, are essential to improve the quality of care and support provided to healthcare recipients with chronic illnesses.

## Figures and Tables

**Table 1:** Characteristics of study participants (*n* = 276).

Variables		N (%)
Age, Years	55.21 SD = 20.3	
Gender	Female	190 (68.8)
	Male	86 (31.2)
Education level	Illiterate	79 (28.6)
	Primary school	33 (12)
	Middle school	27 (9.8)
	Secondary school	49 (17.8)
	University	82 (29.7)
	Postgraduate	6 (2.2)
Marital status	Single	54 (19.6)
	Married	155 (56.2)
	Divorced	11 (4)
	Widowed	56 (20.3)
Employment	Employee	50 (18.1)
	Nonemployee	226 (81.9)
Nationality	Saudi	269 (97.5)
	Non-Saudi	7(2.5)
Region of residence	Central	173 (62.7)
	East	29 (10.5)
	West	50 (18.1)
	North	22 (8)
	South	2 (0.7)
Urban or Rural	Urban	243 (88)
	Rural	33 (12)
Living arrangement	Alone	12 (4.3)
	With spouse	29 (10.5)
	With spouse and children	121 (43.8)
	With older sons/daughter	46 (16.7)
	With family relative	18 (6.5)
	Other	50 (18.1)
Relationship to the caregiver	Spouse	45 (16.3)
	Son or daughter	171 (62)
	Parent	54 (19.6)
	Permeant housekeeper	24 (8.7)
	Temporary housekeeper	11 (4)
	Friend or neighbors	2 (0.7)
	Other relatives	28 (10.1)
Distance between patients and caregiver	Living together	232 (84.1)
	Less than 10 min	14 (5.1)
	11–30 min	17 (6.2)
	More than 30 min	13 (4.7)
Care provided per week on average, hours	≤8	152 (55.1)
	9–19	62 (22.5)
	20–40	62 (22.5)
Length of care	0–3 months	47 (17)
	4–12 months	24 (8.7)
	One to two years	39 (14.1)
	More than two years	166 (60.1)

SD: standard deviation.

**Table 2:** Caregivers’ roles and responsibilities (*n* = 248).

Responses	Never Applicable N (%)	Sometimes Applicable N (%)	Always Applicable N (%)
Obtain medication from the pharmacy	25 (9.1)	82 (29.7)	141 (51.1)
Remind you to take medications	28 (10.1)	82 (29.7)	138 (50)
Prepare pill boxes	55 (20.3)	65 (23.6)	127 (46)
Hand you the medications	53 (19.2)	81 (29.3)	114 (41.3)
Write down a report of falling or any medication side effects	40 (14.5)	86 (31.2)	122 (44.2)
Measure patient’s blood pressure and sugar	49 (17.8)	78 (28.3)	121 (43.8)
Inject medication into patients	112 (40.6)	47 (17)	89 (32.2)
Keep track of medication refill	22 (8)	62 (22.5)	164 (59.4)
Escort you to an appointment	14 (5.1)	59 (21.4)	175 (63.4)
Schedule doctor appointment	32 (11.6)	50 (18.1)	166 (60.1)
Assist you in performing physiotherapy exercise	92 (33.3)	59 (21.4)	97 (35.1)
Prepare special food for you	47 (17)	73 (26.4)	128 (46.4)
Assist you in eating	55 (20.3)	81 (29.3)	111 (40.2)
Assist you in bathing and personal hygiene	137 (49.6)	40 (14.5)	71 (25.7)
Wound ostomy care	118 (42.8)	51 (18.5)	79 (28.6)
Encourage the patients to adhere to a healthy lifestyle	22 (8)	63 (22.8)	163 (59.1)

**Table 3:** Patient’s response regarding challenges encountered during care (*n* = 248).

Responses	Never Applicable N (%)	Sometimes Applicable N (%)	Always Applicable N (%)
Caregiver made an error during medication management (e.g., wrong medication, wrong frequency/dose)	197 (71.4)	44 (15.9)	7 (2.5)
Caregiver stored medication (e.g., insulin) inappropriately	216 (78.3)	27 (9.8)	5 (1.8)
Inappropriate disposal of injections	200 (72.5)	26 (9.4)	22 (8)
Caregiver missed doses	171 (62)	70 (25.4)	7 (2.5)
Caregiver lacks specialized skills needed (e.g., using injection or nebulizers)	178 (64.5)	49 (17.8)	21 (7.6)
Caregivers are not consistently monitoring my condition	184 (66.7)	53 (19.2)	11 (4)
The caregiver does not understand the assigned task/ limited health literacy	165 (59.8)	66 (23.9)	17 (6.2)
Difficulty in communicating due to language barrier	205 (74.3)	27 (9.8)	16 (5.8)
Inconsistent care because caregiver has other responsibilities	149 (54)	69 (25)	30 (10.9)
Lack of empathy	194 (70.3)	41 (14.9)	13 (4.7)
The caregiver has no time	123 (44.6)	115 (41.7)	10 (3.6)
Inconsistent care because I cannot afford a paid domestic worker	172 (62.3)	44 (15.9)	32 (11.6)

## Data Availability

The data will be available for review from the corresponding author upon request.

## Supplementary Materials

The following supporting information can be downloaded at: https://www.mdpi.com/article/10.3390/healthcare13091038/s1, File S1: Study Survey; Table S1: Type of chronic disease and number of medications patients take.
