# Supplementary material for: Assessing the Roles and Responsibilities of Informal Caregivers from the Perspective of Adult Patients in Saudi Arabia: A Cross-Sectional Study
Source: Healthcare (Basel). 2025 May 1;13(9):1038. doi: 10.3390/healthcare13091038 (PMC12071374; doi:10.3390/healthcare13091038)
Supplement: Supplementary file 1 [file healthcare-13-01038-s001.zip › healthcare-3453892-supplementary.pdf]

Supplementary File S1 Study Survey

In this survey a caregiver means a family member or domestic worker who is providing healthcare services to a patient. Care-recipient is an elderly or adult with a temporary or permanent disability, who are unable to perform daily activities and need assistance in managing their health and day-to-day living activities.

Dear Participants
Please answer the following questions to determine If you eligible for this study

1- Please indicate your age

-Less than 18 -More than 18

2- Do you currently live in Saudi Arabia

- Yes -No

3- Do you suffer from temporary or permanent disability or chronic illness?

- Yes -No

4- Do you receive any assistance in managing your health conditions or disability?

- Yes -No

If you answered yes for Q 4, What is the relationship of the person who provides the assistance?

Son/ daughter	Permanent housemaid
Spouse	Temporary housemaid
Parent	Friend / neighbor
Other relative	

If you Answered "No" to any of these questions, close the interview, the patient is not eligible

If you Answered yes to Q 4, what is the main reason for having this assistance

- ☐ Physical inability to manage your health
- ☐ Mental inability to manage your health (depression or anxiety)
- ☐ Having visual and hearing impairment
- ☐ Complicated health condition and multiple medications
- ☐ Lack of timely information and poor communication with HCPs
- ☐ Memory loss or forgetfulness
- ☐ Difficulty in using advanced technology for appointments and refills i.e. SIHI

Patient Demographic Information
**Age**<br>☐18-24 ☐25-34 ☐35-44 ☐45-54<br>☐55-64 ☐65-74 ☐75-84
**Gender**<br>☐Female ☐Male
**Educational Level**<br>☐ illiterate ☐primary ☐secondary ☐university<br>☐Postgraduate
**Marital_status**<br>☐ Single<br>☐ Married<br>☐ Divorced<br>☐ Widowed
**Employment**<br>☐Employee<br>☐ Non employee
**Nationality**<br>☐Saudi ☐Non-saudi
**Region of residence**<br>☐ Central<br>☐East<br>☐West<br>☐North<br>☐South
**Are urban or rural ?**<br>☐Urban ☐Rural
**Family income :**<br>☐ less than 5000SR ☐ 5001-10000SR ☐10001-15000SR ☐15001-20000SR<br>☐ More than 20000SR
**Living arrangement:**<br>☐ Alone<br>☐ With Spouse

☐ With Spouse and children
☐ With older sons\daughters
☐ With family relatives

Hours of care provided per week, on average

0-8 hours
9-19 hours
20-40 hours

Length of care

0-3 months
4-12 months
12-24 months
25-60 months
More than 60 months

Other medical conditions

☐ Obesity
☐ Arthritis
☐ Diabetes
☐ Asthma
☐ High blood pressure
☐ Cancer
☐ Chronic pain
☐ Depression
☐ Urinary Incontinence
☐ other chronic diseases please specify:

Factors that may increase the level of involvement:

Do you use any medications that require specific instruction and require an extra help from the caregiver?
No
Yes, please specify
☐ Inhalers and nebulizers
☐ Injections
☐ Patches
☐ Eye/Ear Drops
☐ Topical medication for hard to reach areas
Other:

How many different medicines do you take daily?
☐ 1-2 medications
☐ 3-5 medications
☐ More than 5 medications

Roles and responsibilities of caregivers (please check all that applies)

Obtain medications from pharmacy
Remind you to take medications
Preparing pill boxes
Handing you the medications
Write down\report falling or any side effect from medication
Measure patients' blood pressure and sugar
Inject medications to patient
Keeping track of medication refills
Escorting you to an appointment
Scheduling doctor appointment
Assist you in performing Physiotherapy exercises
Prepare special food for you
Assist you in feeding
Assist you in bathing and personal hygiene
Wound /Ostomy Care
Encourage the patient to adhere to a healthy life style
Others

Which of the following problems have you encountered during care provided by caregiver? (please check all that applies)

None

**Drug related problem**
Caregiver made errors during medication management e.g. wrong medicine or wrong frequency\dose
Caregivers stored medication inappropriately e.g. insulin
Inappropriate disposal of injections
Caregiver missed doses
Caregiver lacks specialized skill needed e.g. using injections or nebulizers
Caregiver are not consistently monitoring my condition
**Caregiver related issues**
Caregiver doesn’t understand the assigned tasks\ Caregiver limited health literacy
Difficulty communicating due to language barrier
Inconsistent care as caregiver has other responsibilities
Lack of empathy
Caregiver doesn’t have time
Inconsistent care as I cannot afford a paid domestic worker

Table S1. Type of chronic disease and number of medications patients take

Variable	N (%)
**Chronic diseases***	
Diabetes	173 (62.7)
Hypertension and cardiovascular diseases	127 (46)
Asthma	39 (14.1)
Rheumatoid arthritis	82 (29.7)
Cancer	8 (2.9)
Anxiety or depression	36 (13)
Chronic pain	49 (17.8)
Incontinence	25 (9.1)
**Number of medications**	
1-5	154 (55.8)
6-10	59 (21.4)
11-15	16 (5.8)
16-20	1 (0.4)
**Medication and treatments that require caregiver assistance**	189 (68.5)
Inhalers	39 (14.1)
Injections	77 (27.9)
Patches	19 (6.9)
Eye or ear drops	53 (19.2)
Local medications for hard-to-reach area	40 (14.5)

*The number is greater than expected because some respondents may have had more than one answer.
